# Potential of Nano-Antioxidants and Nanomedicine for Recovery from Neurological Disorders Linked to Long COVID Syndrome

**DOI:** 10.3390/antiox12020393

**Published:** 2023-02-06

**Authors:** Thelma Akanchise, Angelina Angelova

**Affiliations:** Université Paris-Saclay, CNRS, Institut Galien Paris-Saclay, 91400 Orsay, France

**Keywords:** antioxidant-delivery nanosystems, lipid nanocarriers, oxidative stress, ROS, neuroinflammation, neurodegeneration, neurological long COVID-19

## Abstract

Long-term neurological complications, persisting in patients who cannot fully recover several months after severe SARS-CoV-2 coronavirus infection, are referred to as neurological sequelae of the long COVID syndrome. Among the numerous clinical post-acute COVID-19 symptoms, neurological and psychiatric manifestations comprise prolonged fatigue, “brain fog”, memory deficits, headache, ageusia, anosmia, myalgias, cognitive impairments, anxiety, and depression lasting several months. Considering that neurons are highly vulnerable to inflammatory and oxidative stress damages following the overproduction of reactive oxygen species (ROS), neuroinflammation and oxidative stress have been suggested to dominate the pathophysiological mechanisms of the long COVID syndrome. It is emphasized that mitochondrial dysfunction and oxidative stress damages are crucial for the pathogenesis of neurodegenerative disorders. Importantly, antioxidant therapies have the potential to slow down and prevent disease progression. However, many antioxidant compounds display low bioavailability, instability, and transport to targeted tissues, limiting their clinical applications. Various nanocarrier types, e.g., liposomes, cubosomes, solid lipid nanoparticles, micelles, dendrimers, carbon-based nanostructures, nanoceria, and other inorganic nanoparticles, can be employed to enhance antioxidant bioavailability. Here, we highlight the potential of phytochemical antioxidants and other neuroprotective agents (curcumin, quercetin, vitamins C, E and D, melatonin, rosmarinic acid, N-acetylcysteine, and Ginkgo Biloba derivatives) in therapeutic strategies for neuroregeneration. A particular focus is given to the beneficial role of nanoparticle-mediated drug-delivery systems in addressing the challenges of antioxidants for managing and preventing neurological disorders as factors of long COVID sequelae.

## 1. Introduction

The management of the post-acute coronavirus SARS-CoV-2 infection presents current concerns for society as the global pandemic has massively affected health, the economy, education, and employment since the outbreak of COVID-19 in 2019 [1,2,3,4]. Several reports affirmed that many patients who survived the COVID-19 illness continue to exhibit neurological COVID symptoms and fail to revert to their regular daily routines [3,4,5]. Such a post-COVID-19 condition has been classified as “long COVID”, “long-haul COVID”, “post-acute COVID syndrome”, “chronic COVID”, and post-acute sequelae of SARS-CoV-2 (PASC) [5,6,7,8,9,10,11,12]. It has been admonished that “long COVID could be the next public health disaster in the making” [13]. The long COVID syndrome encompasses various clinical symptoms that linger for more than 12 weeks and may last over the years [3,5,10,11,12]. This syndrome is a multi-organ disease that affects the central nervous system (CNS) and the peripheral nervous system (PNS) in addition to other systems, including the pulmonary, cardiovascular, endocrine, renal, gastrointestinal, and immunological systems [2,3,4,6,8,9,10,11,12,14,15,16,17,18]. The National Institute of Health (NIH) has pointed out the most common symptoms associated with long COVID, namely headaches, depression, anosmia, cognitive impairments, shortness of breath, “brain fog”, fever, and gastrointestinal symptoms [5,6,8]. More severe cases include myalgic encephalitis/chronic fatigue syndrome (ME/CFS) and stroke, indicating that the neurological symptoms are the core aspects of long COVID [9,10,11,12,13,14].

The SARS-CoV-2 virus invades the CNS by binding to the angiotensin-converting enzyme-2 (ACE2) receptor, which is widely distributed in the epithelial cells of lungs and the endothelial cells of the blood–brain barrier (BBB) [19,20]. The neuroinvasive potential of the coronavirus is revealed by the provoked neuroinflammation and neuronal demyelination of the CNS, cellular apoptosis, metabolic imbalances, and various coagulopathies and endotheliopathies that induce hypoxic-ischemic neuronal injury or blood–brain barrier dysfunction [1,5,8,9,16,18].

**Figure 1:**
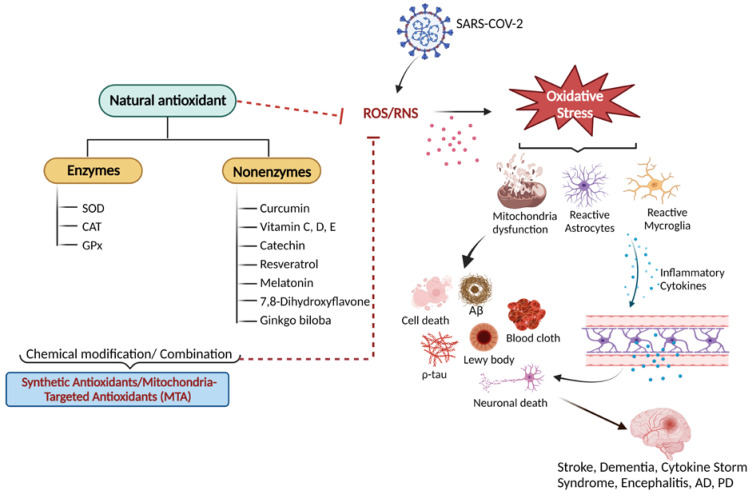
An overview of natural antioxidant systems and their role in scavenging ROS/RNS: Excessive ROS/RNS production triggers oxidative stress that causes mitochondrial dysfunction accompanied by the activation of astrocytes and microglia. Glial cell activation is associated with the release of inflammatory cytokines and chemokines, ultimately causing cellular apoptosis and neuronal death.

Hyper-inflammation and oxidative stress have been suggested as the key factors underlying the pathophysiological pathways of long COVID [21,22,23] (Figure 1). It is recognized that the coronavirus infection compromises mitochondrial regulation and causes a decrease in adenosine triphosphate (ATP) synthesis and the activation of nicotinamide adenine dinucleotide phosphate (NADPH) oxidase. This contributes to the production of reactive oxygen species (ROS). Virus invasion also leads to inflammatory responses in the brain by triggering the release of cytokines, including interleukin (IL-10), tumor necrosis factor-alpha (TNF-α), and interferon-gamma (INF-γ). This cascade goes a long way to further increase mitochondrial ROS production through the upregulation of mitochondrial genes and the modulation of the electron transport chain (ETC) [23,24,25,26].

Evidence has demonstrated that oxidative stress caused by excessive ROS production, accompanied by hypoxia, contributes to the death of dopamine-containing neurons (DCNs) via apoptosis (Figure 1) and subsequently leads to Parkinson’s disease (PD), a progressive neurodegenerative disorder [26]. Multiple epidemiological and clinical research indicates a substantial correlation between the SARS-CoV-2 infection, long COVID, and the onset of progressive neurodegenerative diseases that include Alzheimer’s disease (AD), prion disease (PrD) and PD, thus rendering it a cause for alarm [26,27].

There is still no cure for the neurological sequelae of COVID-19. During acute COVID-19, patients have been treated with corticosteroids, antibiotics, and antiviral drugs [1]. Antioxidants are attracting scientific interest in the therapy and prevention of COVID-induced neuronal damage [4,28]. Examples of antioxidants include vitamins C, E and D, curcumin, quercetin, melatonin, and rosmarinic acid [4,7,28,29,30,31,32,33,34]. These molecules have advantages in counteracting the oxidative-stress effects by scavenging ROS, combating the cytokine storm, and providing neuroprotection, as summarized in Figure 1.

Antioxidants with poor bioavailability and low water solubility have limited therapeutic usage [35,36]. To solve these challenges, nanomedicine-based delivery systems, lipid-based nanoparticles, polymeric particles, and other nanostructured assemblies are gaining much attention as a promising field for drug delivery and a valuable tool for future medicine [37,38,39,40]. Given that they can enhance pharmacokinetics, it is feasible to obtain the proper targeting and lower toxicity of these medicinal compounds [40].

Here, we present an overview of the current knowledge regarding the long-term neurological effects of COVID-19 and suggest therapeutic approaches involving nanoantioxidants and nanomedicine-based strategies in regeneration therapies of coronavirus-induced neurodegenerative cascades.

### 1.1. Pathophysiological Mechanism Underlying Neurological Manifestation of Long COVID

In the context of long COVID, aside from neuroinflammation, other neuropathological processes such as prothrombotic, hypoxic, metabolic, and apoptotic cascades prevail in the propagation of neurological manifestations [1,6]. According to clinical facts, neurological symptoms encountered by long COVID patients (including hyposmia, mood disorders, cognitive impairment, sleep disorders, and dysautonomia) have been linked to the dysfunction of ‘ACE2-rich’ brain areas involving the olfactory bulb, the amygdala, the hippocampus, the middle temporal gyrus, the posterior cingulate cortex, and the brainstem [15,41,42].

**Figure 2:**
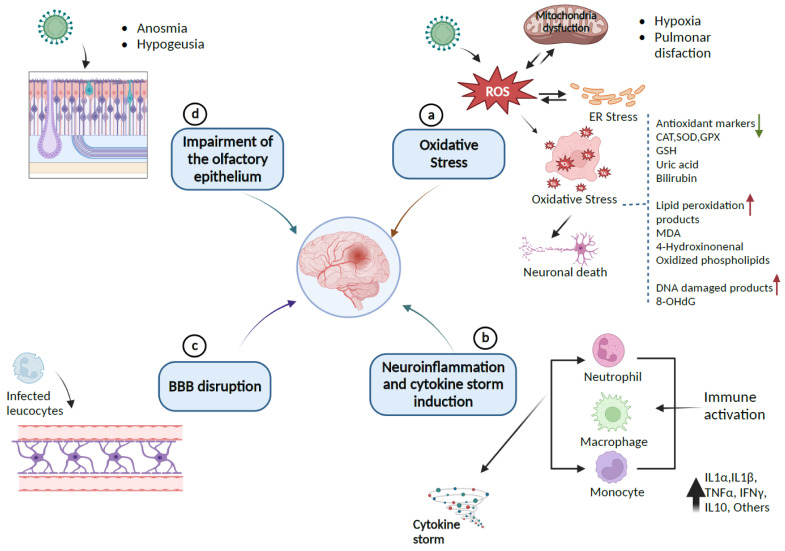
Pathway of SARS-CoV-2-induced neurological damages: (a) SARS-CoV-2 provokes the release of ROS, which leads to endoplasmic reticulum stress (ER) or oxidative stress that can damage lipids, proteins, and DNA biomolecules in the cells and can downregulate the expression of antioxidant proteins CAT, SOD, GPx, GSH, and the levels of uric acid and bilirubin as causative factors for neuronal cell death. (b) Immune response to inflammation triggers inflammatory markers involving cytokines and chemokines that can initiate cytokine storm syndrome. (c) Infected leukocytes can infect the central nervous system by crossing and disrupting the BBB. (d) SARS-CoV-2 enters the CNS through the olfactory epithelium, which expresses the ACE2 receptor. The resulting infection causes neuronal death and olfactory dysfunction. Up and down arrows indicate the upregulation and downregulation of biomarkers, respectively (Created with BioRender).

A recent positron-emission tomography (PET) scan of 35 patients with long COVID revealed bilateral hypometabolism in the bilateral rectal/orbital gyrus, including the olfactory gyrus and the right temporal lobe, involving the amygdala and the hippocampus [43]. The involvement of the brainstem and the cerebellum highlights the vulnerability of the CNS and underlies the neurological manifestations of long COVID, which are comparable to postural orthostatic tachycardia syndrome (POTS) and myalgic encephalomyelitis or chronic fatigue syndrome (ME/CFS) [43,44,45]. Figure 2 presents a scheme of the possible pathways by which SARS-CoV-2 causes neuronal injury.

### 1.2. Role of Antioxidants in Neuroprotection from Neurological Long COVID Sequelae

To address the challenges of the long COVID syndrome, the National Institute for Health and Care Excellence (NICE) has issued a diagnostic approach and treatment guidelines to promote the management of long COVID with a peculiar focus on neurological manifestations [46,47,48,49,50]. Even though more than 200,000 papers on COVID-19 have been published in less than three years, there is still a striking gap in the literature on specific treatment recommendations for neurological long COVID sequelae.

**Figure 3:**
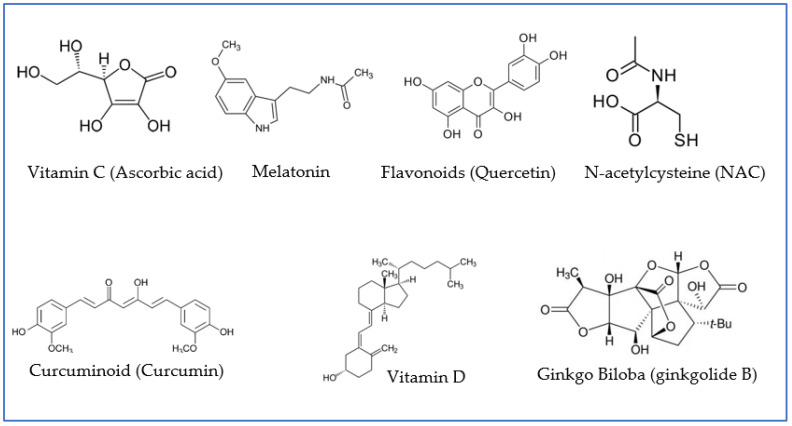
Examples of antioxidant agents with neuroprotective properties that have been proven to play a role in preventive and/or adjuvant therapies for patients infected with COVID-19. Quercetin is presented as an example of flavonoids; ginkgolide B, as an example of terpenoid, derivative of ginkgo biloba; and curcumin, as an example of curcuminoid.

Here, we highlight some therapeutic agents that may significantly help to ameliorate long-term-COVID conditions. Amongst these are antioxidant compounds or agents endowed with antioxidant properties (Figure 3). They have demonstrated an improved prognosis of COVID-19 patients via different mechanisms, mainly by reducing inflammation [28,51]. In many cases, these compounds share multiple mechanisms of action, which may render them more efficient in exerting broad-spectrum protective effects. In a synergic fashion, their neuroprotective properties may be enhanced by complementation with antioxidants with different mechanisms of action. Most antioxidant compounds are molecules from natural sources or of dietary origin (nutraceuticals). In the latter case, a balanced diet and supplementation with proper nutrients can contribute to the prevention, treatment, and management of COVID-19 and its associated neurological sequelae.

## 2. Nanodelivery Systems for Development of Antioxidant-Based Nanomedicines against the Neurological Sequelae of SARS-CoV-2

**Figure 4:**
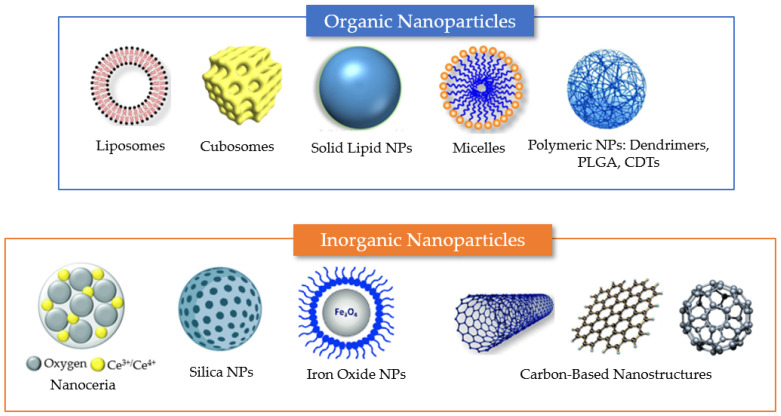
Summary of different nanosystem types employed for drug encapsulation to overcome the limitations of antioxidant delivery in neuronal regeneration from post-COVID-19 damages. Examples of organic delivery vehicles include liposomes, cubosomes, solid lipid nanoparticles, micelles, and polymeric nanoparticles. Examples of inorganic nanoparticles include nanoceria, mesoporous silica nanoparticles, and iron oxide NPs. Carbon-based materials, which lack carbon–hydrogen bonds typical of organic compounds, include various types of nanostructures (e.g., carbon nanotubes, graphene nanosheets, and fullerenes). The properties of the different nanocarriers are described in the text.

Nanomedicine-based therapies against the neurological sequelae of long COVID will require developing nanoscale delivery systems for the efficient use of antioxidants. In terms of drug delivery, antioxidants display several limitations, including (i) low permeability into the CNS due to the presence of physiological barriers such as the BBB or the spinal–blood barrier (SBB) [52]; (ii) low bioavailability associated with insolubility or instability [53]; (iii) chemical and physical barriers in the gut such as the acidic pH of the stomach, the intestinal mucosal lining, and the selectively permeable membranes of enterocytes [54]; and (iv) rapid metabolism [55]. Significant efforts have been made to improve the efficacy of antioxidant agents using various drug-delivery approaches. Figure 4 shows examples of nanocarriers fabricated with natural or synthetic compounds, e.g., lipids, polymers, or inorganic materials. In general, formulating antioxidants in nanocarriers can enhance their efficacy due to a higher stability upon encapsulation and an improved transport to the CNS compared with free antioxidant compounds.

### Nanoparticle Types as Antioxidant Carriers

Nanoparticles (NPs) can be categorized as (i) purely organic compound NPs or (ii) inorganic NPs, based on the elements that built up their structure (Figure 4) [56]. The majority of organic materials are biocompatible, biodegradable, and non-toxic. However, inorganic materials are characterized by their smaller particle size, stability, controlled tunability, higher permeability, efficient antigen loadings, and triggered-release profile.

Organic molecule-based nanosystems developed as delivery vehicles of antioxidants include liposomes, micelles, and polymeric nanoparticles. Liposomes, for example, are lipid-based nanomaterials that consist of an aqueous core surrounded by a phospholipid bilayer. Depending on the hydrophobic/hydrophilic balance and molecular shapes, the amphiphilic structures enable the formation of thermodynamically stable vesicles [57]. Amphiphilic molecules can also self-assemble to form micelles, which contain hydrophilic (polar) headgroups and hydrophobic (nonpolar) tails. In aqueous media, micelles are typically assembled with the polar part facing the exterior and the nonpolar region constituting the core.

Solid lipid nanoparticles (SLNs) are widely known transporters of chemotherapeutic agents to the CNS [37]. These matrix-type lipid particles, usually consisting of fatty acids or mono-, di-, or triglycerides, remain with a solid core at body temperature. Cubosomes are liquid crystalline nanostructures created from cubic-phase-forming lipids, such as monoolein and phytantriol [29,35,38], which have the unique ability to disperse into nanoparticles that are stable upon dilution [57,58,59]. Liposomes and cubosomes can encapsulate hydrophobic drugs in the lipid bilayer membranes and hydrophilic compounds in the aqueous compartments [29,35,37].

Polymeric nanoparticles are particles with sizes between 5 and 1000 nm. They can be loaded with active substances, which are either surface-adsorbed onto the polymeric matrix core or entrapped within. The various biodegradable polymers commonly used in the fabrication of polymeric nanoparticles include poly(lactide) (PLA), poly(lactide-co-glycolide) (PLGA) copolymers, poly (ɛ-caprolactone) (PCL), and poly(amino acids), as well as some natural polymers such as alginate, chitosan, and gelatin [30,60,61,62].

Inorganic nanoparticles are primarily known for their use in diagnostic and theranostic applications. Iron oxide nanoparticles (IONPs) are one type of inorganic NPs that has been extensively employed for therapeutic and diagnostic imaging since the 1960s. Gold nanoparticles (AuNPs) have been widely studied due to their biocompatibility and the ease of controlling their size distribution and shape. AuNPs can take a variety of shapes, including spheres, nanorods, and cubes, among others. Other inorganic nanoparticles are nanoceria, silica nanoparticles (SiNPs), particularly mesoporous silica nanoparticles (MSNPs), and iron oxide NPs [63,64,65] (Figure 4).

## 3. Organic-Molecule-Based Nanoparticulate Delivery Systems

### 3.1. Curcumin-Loaded Nanoparticles

In light of the COVID-19 pandemic, at least three types of curcumin-based nanotechnological products are available on the market in the form of liposomes (Lipocurc™), polymeric nanoparticles (Nanocurc™), or nanomicelles (Sinacurcumin^®^) [66]. Sinacurcumin^®^ (40 mg, four capsules daily for 14 days) has been shown to reduce mortality by decreasing the cytokine storm [67]. Nanoparticulate formulations (e.g., curcumin-loaded Se-PLGA nanospheres) have proven their potency to improve cognition, inhibit the aggregation of Aβ, and reduce depressive-like behaviour and oxidative stress in AD models [68].

Curcumin is one of the most-marketed antioxidant compounds with a promising nutraceutical profile and a safety tolerated dose of up to 12 g/day [29,66,69,70]. Zahedipour et al. have emphasized the remarkable effects of curcumin in the treatment of COVID-19 [70]. The pleiotropic effects of the phytochemical against viruses are related to its ability to interact with various molecular targets by modulating various signaling cascades, which are relevant for virus replication, by attenuating NF-κB and PI3K/Akt signaling as well as regulating the expression of both pro- and anti-inflammatory proteins such as IL-6, IL-8, IL-10, and COX-2. In this way, curcumin impacts the apoptosis of polymorphonuclear neutrophil cells (PMNs), an abundant immune-system cell type. In an animal model of stroke, Jiang et al. have shown that curcumin treatments result in an essential total decrease in the infarct volume, an improved neurological deficit, and a reduced mortality in a dose-dependent manner [71].

In animal models of depression, curcumin has been found to normalize the levels of dopamine, noradrenaline, and 5-hydroxyindoleacetic acid in the frontal cortex of rats. This outcome indicates that curcumin may act as a potent antioxidant against depression [72,73]. Regarding anosmia and ageusia, a case series has revealed a rapid and effective recovery of taste and smell in two subjects infected with COVID-19 following the ingestion of a 1000 mg dosage of a turmeric supplement that contained 95% curcuminoids [74].

**Figure 5:**
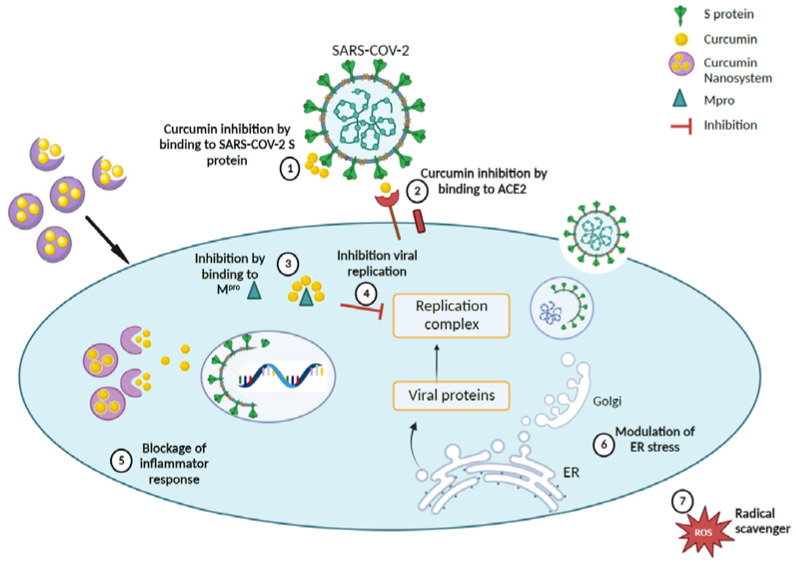
Proposed therapeutic mechanism for curcumin-loaded nanosystems in accordance with literature findings [67,68]. (1), (2), and (3) Curcumin nanocarriers inhibit SARS-CoV-2 by delivery of curcumin to bind to the S protein, ACE2 receptor, and Mpro of the coronavirus, thereby (4) impeding the virus replication complex. (5) Curcumin nanocarriers can also block inflammatory response by (6) modulating ER stress and (7) scavenging ROS. (Created with BioRender).

An intriguing study showed that curcumin-loaded insulin d-α-tocopherol succinate (INVITE) micelles enhanced the ability of mesenchymal stromal cells (MSCs) to boost neuronal protection and replace dead motor neurons in the spinal cord. This ground-breaking strategy has been demonstrated to have considerable potential for the treatment of ALS [75]. The effects of curcumin-loaded nanoparticles on the neurological effects of SARS-CoV-2 are revealed in Figure 5.

### 3.2. Curcumin Nanoconjugates

Peptide (B6)-conjugated curcumin-loaded PLGA-PEG-B6 nanoparticles have been established to decrease hippocampal Aβ burden in APP/PS1 mice as revealed by immunofluorescence and immunohistochemistry results [76]. Drug distribution into the CNS is essentially hampered by the relative impermeability of the BBB due to the tight junctions between cerebral microvascular endothelial cells, which also play a significant role in brain homeostasis. Receptors with high endothelial cell expression, including the insulin, transferrin, and integrin receptors, are of particular interest for receptor-mediated transport (RMT) because of their ability to increase the efficiency and specificity of brain delivery [77]. The transferrin receptor (TfR), one of these different receptors, has been the subject of much research for BBB targeting during the past ten years [78]. Nevertheless, further developments are still needed to overcome drawbacks related to immunological responses, NP synthesis methods, and NP stability. The B6 peptide (CGHKAKGPRK), a promising targeting ligand of TfR, has been produced from a phage display library as a replacement for the protein transferrin in drug delivery to the brain [79,80].

In a related study, Yin et al. created sialic acid (SA)-modified selenium (Se) nanoparticles conjugated with the B6 peptide (B6-SA-SeNPs), which had high permeability across the BBB and successfully prevented Aβ peptides from aggregating and disaggregated the preformed Aβ fibrils into non-toxic amorphous oligomers [81].

**Figure 6:**
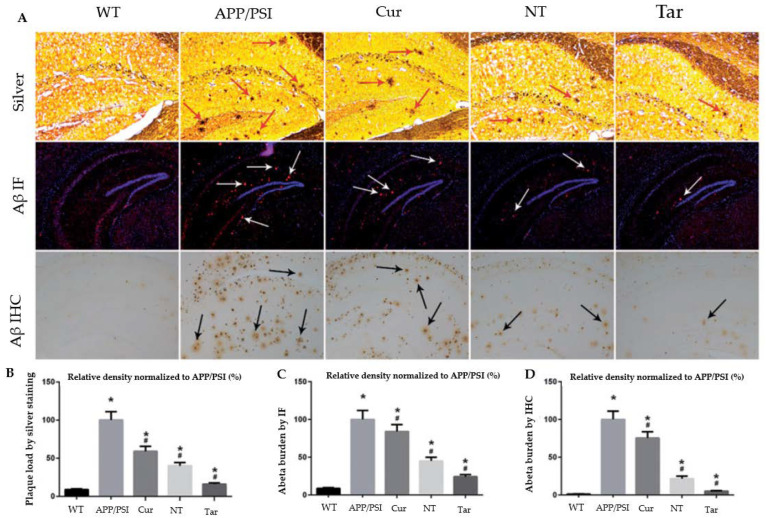
Capacity of Cur-loaded PLGA-PEG-B6 nanoparticles to decrease hippocampal Aβ burden in APP/PS1 mice. The AD animal model (APP/PS1) received saline, free Cur drug, PLGA-PEG/Cur nanoparticles (NT), and PLGA-PEG-B6/Cur nanoparticles (Tar), after which (**A**) Bielschowsky silver staining (red arrows) and immunofluorescence (IF, white arrows) or immunohistochemistry (IHC, black arrows) of Aβ in brain sections were performed. (**B**–**D**) The relative density of Aβ plaque in each group was compared using silver staining, Aβ IF, and Aβ ICH, respectively. The experimental findings show Aβ plaque deposition in APP/PS1 animals compared with wild-type mice. The Cur and NT groups experienced a marginal reduction, whereas the Tar group showed the most substantial reduction in Aβ plaques. (* *p* < 0.05; # *p* < 0.05) Adapted with permission from [76] under Open Access Creative Commons Attribution License. Copyright {2018} Informa UK Limited (Taylor & Francis Group).

Figure 6 shows the reduction in the Aβ plaque deposition in nanoparticle-treated APP/PS1 animals compared with wild-type mice. According to these findings, PLGA-PEG-B6/Cur nanoparticles may be potentially effective for AD therapies when focusing on Aβ pathophysiology in neurological and neuropsychiatric manifestations [76].

### 3.3. NAC-Loaded Nanoparticles

N-acetylcysteine (NAC) is a sulfhydryl-containing compound with mucolytic properties (Figure 3). It functions as a precursor of the antioxidant glutathione and the amino acid L-cysteine [82,83]. The ability of NAC to block certain signaling pathways, such as the c-Jun N-terminal kinase, p38 MAP kinase, SAPK/JNK, and c-Fos pathways, as well as NF-κB, and to regulate cytokine synthesis (anti/pro-inflammatory effect) and antiapoptotic genes is also supported by a number of works [84,85,86].

In Parkinson’s disease, thiol-containing compounds such as NAC exert a protective action by inhibiting dopamine-induced cell death [87]. NAC has partially protected the mouse brain against cadmium-induced neuronal apoptosis by inhibiting the ROS-dependent activation of the Akt/mammalian target of the rapamycin (mTOR) signaling pathway [87]. NAC is a potential candidate for treating neuropathic pain, which has occurred in up to 2.3% of hospitalized patients with COVID-19 [88], as well as ME/CFS, by providing neuroprotection against oxidative stress and replenishing the cortical GSH reserves [89,90]. In healthy BV2 murine microglia, hydroxyl-terminated polyamidoamine dendrimers containing the antioxidant NAC reduced oxidative stress compared with free N-acetylcysteine [91].

**Figure 7:**
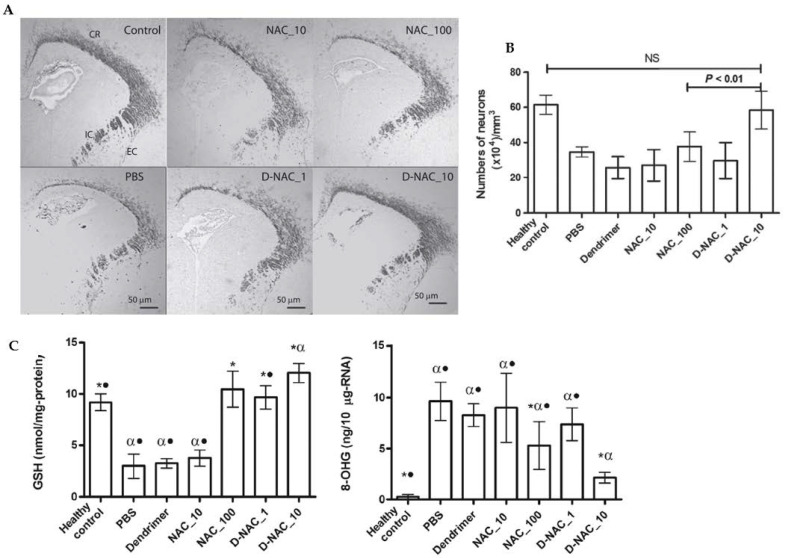
Effects of a NAC-based nanosystem on myelination and neuronal injury in a dendrimer-based therapy (D-NAC) against neuroinflammation and cerebral palsy (CP). (**A**) Confocal microscopy results were obtained with brain sections harvested and stained for myelin basic protein (MBP) using DAB staining. The images show a decrease in the MBP staining in the corona radiata, internal capsule, and external capsule following day 5 of life in endotoxin kits treated with PBS compared with healthy controls. (**B**) Microtubule-associated protein 2 (MAP2) labeling was used to detect mature neurons in the caudate area of the basal ganglia. Treatment with D-NAC (10 mg/kg) significantly enhanced the number of neurons (*p* < 0.01) compared with those of healthy controls. (**C**) Determination of the amounts of 8-hydroxyguanosine (8-OHG) and GSH to assess oxidative injury and inflammation in CP and healthy rabbits. The markers of oxidative injury were compared in the brain’s periventricular region (PVR) after treatment of CP kits with D-NAC 1, D-NAC 10, and free drug at high concentrations (NAC 100). (* *p* < 0.05 when compared to PBS; α *p* < 0.01 when compared to control and • *p* < 0.01 when compared to D-NAC_10) Adapted with permission from [92]. Copyright {2018} Science-HHS Public Access (PubMedCentral).

A dendrimer-based therapy (D-NAC) for neuroinflammation and cerebral palsy (CP) was developed using NAC as a drug [92]. In this work, newborn babies with CP were randomly administered with NAC at concentrations of 10 mg/kg (NAC_10), 100 mg/kg (NAC_100), D-NAC of 1 mg/kg (D-NAC_1), D-NAC of 10 mg/kg (DNAC_10), dendrimer alone (control), or PBS (negative control). Confocal microscopy results (Figure 7) showed a decrease in myelin basic protein (MBP) staining in the corona radiata, internal capsule, and external capsule following day 5 of life in endotoxin kits treated with PBS compared with healthy controls. A significant increase in myelin staining equivalent to the expression levels of healthy controls was seen in the kits treated with D-NAC at 10 mg/kg (D-NAC_10). In contrast, free antioxidants at a concentration of even 100 mg/kg (NAC_100) were less effective than D-NAC_10. Markers of oxidative injury in the brain’s periventricular region (PVR) were used to identify oxidative injury and inflammation. After therapy, the amounts of 8-hydroxyguanosine (8-OHG) and GSH were assessed in healthy and CP rabbits. The treatment of the CP kits with D-NAC 1, D-NAC 10, and the highest dose of free medication (NAC 100) resulted in a rise in the GSH levels as compared with those of the healthy control kits. However, treatment with NAC_10 and dendrimer alone had no impact. This fact suggests that NAC is released from the brain’s dendrimer conjugate. Even at the highest dose, D-NAC 10 significantly decreased the levels of 8-hydroxyguanosine (8-OHG), an early and sensitive marker for RNA oxidation in various neurodegenerative diseases [92]. The treatment with D-NAC (10 mg/kg) nanosystem significantly enhanced the number of neurons as compared with the healthy controls.

### 3.4. Taxifolin Nanocomplexes

Taxifolin (TAX: 3,3′,4′,5,7-pentahydroxy flavanone or dihydroquercetin) is a vital bioflavonoid polyphenolic antioxidant commonly found in fruits, vegetable oils, red wine, tea, Siberian larch, and onions. In terms of the chemical structure of the compound, the distribution of the hydroxyl groups among the three flavonoid rings (A, B, and C) is the same for taxifolin and quercetin. However, the two molecules differ in the presence or absence of a C-2 or C-3 double bond in the C-ring. Other derivatives of taxifolin include neoastilbin, astilbin, isoengeletin, isoastilbin, engeletin, taxifolin 7-O-glucoside, taxifolin 3-O-glucoside, taxifolin-7-O-rhamnopyranosid and taxifolin-3-O-rhamnopyranoside [93]. Taxifolin and its derivatives have shown to offer pharmacological benefits, including antioxidant, anti-inflammatory, antiviral, antibacterial, and enzyme-inhibitory properties. For instance, taxifolin exhibits an immediate neuroprotective effect, principally by decreasing the generation of ROS in the inhibitory population of GABA neurons. Furthermore, taxifolin can affect gene expression, which modulates cell survival and death. When administered at doses of 0.1 and 1.0 g/kg, taxifolin reduced infarction by 42% ± 7% and 62% ± 6%, respectively, in a rat ischemia-reperfusion (I/R) model. This was followed by a significant reduction in adduct formation of malondialdehyde and nitrotyrosine, two markers for oxidative tissue damage [94]. In a concurrent treatment, taxifolin and cilostazol synergistically inhibited amyloidogenesis by suppressing P-JAK2/P-STAT3-coupled NF-κB-linked BACE1 expression via the upregulation of SIRT1 in activated N2a Swe cells geared towards the intervention of AD [95,96].

**Figure 8:**
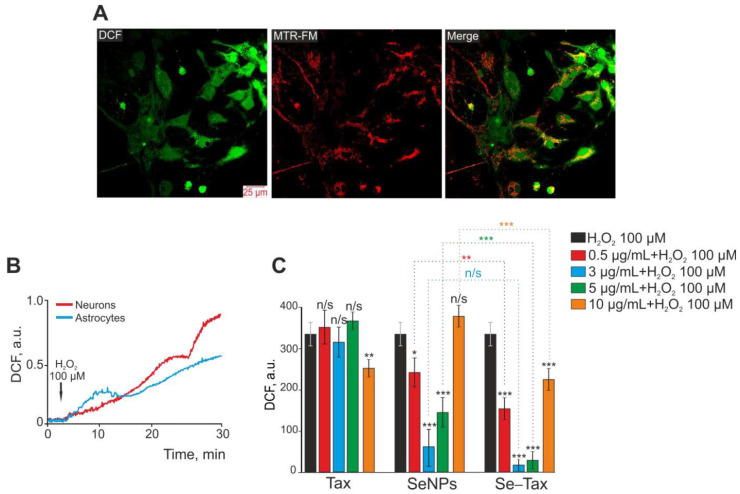
Taxifolin (TAX), selenium nanoparticles (SeNPs), and selenium–taxifolin nanocomplex (Se–TAX) pretreatments of cerebral cortex cells for 24 h have a dose-dependent impact on ROS generation induced by 100 μM H_2_O_2_. (**A**) Confocal fluorescence images with simultaneous labeling of cerebral cortex cells with a DCF probe (to quantify ROS generation), a mitochondrial probe (MitoTracker Red FM, MTR-FM), or a combination image (Merge). (**B**) Generation of ROS in cerebral cortex neurons (red curve) and astrocytes (blue curve) after adding 100 μM H_2_O_2_ to the cell culture. (**C**) Impact of TAX, SeNPs, and Se–TAX, at varying doses, on the H_2_O_2_-induced ROS generation after 24 h cell incubation (measurements performed using a microplate reader). Adapted with permission from [97]. (*** *p* < 0.001; ** *p* < 0.01 and * *p* < 0.05. n/s—insignificant differences). Copyright {2022} Science-HHS Public Access (PubMedCentral).

Targeted delivery has been realized by nanocomplexes of selenium nanoparticles (SeNPs) with taxifolin (Se–TAX). In a recent work (Figure 8), Varlamova et al. developed selenium–taxifolin nanocomplexes to reduce ROS in neurons and astrocytes exposed to exogenous H_2_O_2_ under ischemia-like circumstances [97]. This mechanism was demonstrated by activating certain antioxidant enzymes and inhibiting ROS-generating systems during OGD/reoxygenation. While free TAX molecules and “naked” SeNPs were less efficient in controlling the cellular redox state, Se–TAX decreased the concentration of cytosolic Ca^2+^ ([Ca^2+^]i) rise and hyperexcitation. The nanocomplex activated protective genes while simultaneously suppressing the production of pro-inflammatory and proapoptotic proteins [97]. There is expanding research towards a better understanding of the role of calcium during viral infections, particularly in SARS-CoV-2. In this direction, the suppression of calcium transport across membranes and inside cells is a perspective target site that may alter the SARS-CoV-2 infection and potentially benefit severe COVID-19 courses [98]. Given these data (Figure 8), it has been inferred that the Se–TAX nanocomplex controls Ca^2+^ dynamics and has anti-apoptotic properties [97].

### 3.5. Other Organic Nanovectors

Antioxidants such as polyphenols have shown a capacity to interfere with various stages of the coronavirus entry as well as inhibitory activities against viral components, rendering them potentially suitable to counteract the SARS-CoV-2 infection [99]. Computational studies have indicated that flavonoids, which are a class of polyphenols including quercetin (Figure 3), baicalin, luteolin, hesperetin, gallocatechin gallate (GCG), epigallocatechin gallate (EGCG), naringenin, cyanidin, genistein, kaempferol, luteolin-7-glucoside, apigenin-7-glucoside, catechin, taxifolin, and rutin, can exert an inhibitory activity against SARS-CoV-2 by binding to essential proteins involved in the coronavirus infection cycle such as Mpro, PLpro, 3CLpro, and NTPase/helicase [28,100,101,102,103,104].

Debnath et al. have demonstrated that nano-quercetin could exhibit an anti-amyloidogenic activity at low quercetin concentrations, thereby preventing polyglutamine aggregation in a cell model of Huntington’s disease [105]. It has been established that COVID-19 causes a disruption of the cholinergic system by binding to nicotinic acetylcholine receptors (nAChRs), which results in a change in acetylcholine activity [106]. In this regard, the established protective role of quercetin and rutin against scopolamine-induced cognitive impairment in zebrafish appears to be promising in the enhancement of cholinergic neurotransmission [107]. Accordingly, Palle and colleagues showed that quercetin nanoparticles (NQCs) significantly reduced MDA and AchE levels while increasing CAT and GSH in a scopolamine-induced rat model of amnesia, demonstrating that it has an activity similar to that of an acetylcholinesterase inhibitor such as rivastigmine [108]. In a separate study, the co-encapsulation of epigallocatechin-3-gallate and acetyl acid (EGCG/AA NPs), which was orally administered to an APPswe/PS1dE9 mice model for Alzheimer’s disease, led to an upregulation of synaptophysin (SYP) and influenced neuroinflammation, Aβ plaque burden, and the cortical levels of both soluble and insoluble Aβ_(1–42)_ peptides, leading to an improvement in learning and memory [109]. Moreover, it has been reported that 4-hydroxyisophthalic acid (4-HIA) encapsulated PLGA-NPs significantly decreased the cytotoxicity of H_2_O_2_ in PC12 cells when compared with non-encapsulated 4-HIA.

Yang et al. have demonstrated the beneficial effect of PLGA-PEG-Fucoxanthin nanoparticles in improving cognitive performance and transport through the CNS [110]. In an animal model of AD, resveratrol-loaded NPs decreased the levels of matrix metalloproteinase-9 (MMP-9) in cerebrospinal fluid, highlighting that resveratrol limits brain permeability, the infiltration of leukocytes, and other inflammatory agents. Resveratrol presents a therapeutic interest because it modulates neuroinflammation and induces adaptive immunity [111].

## 4. Inorganic Nanoparticles and Nanocarriers

### 4.1. Ceria Oxide Nanoparticles

**Figure 9:**
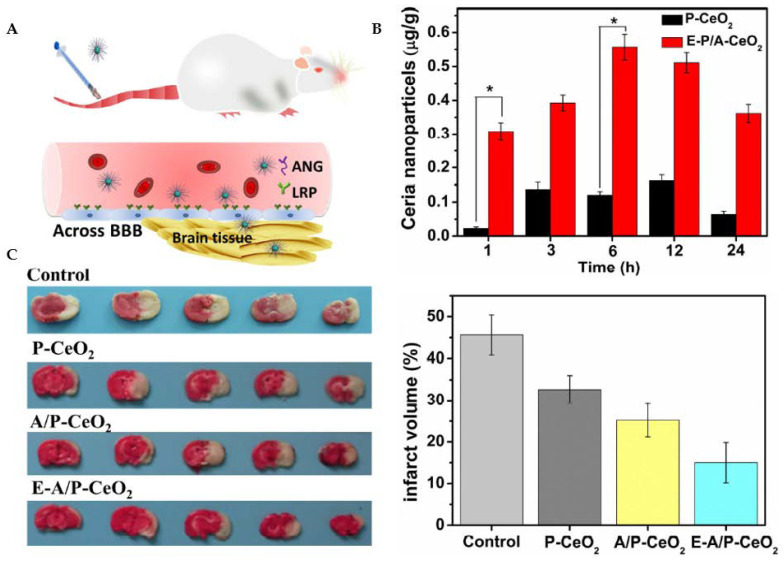
Ceria nanoparticles for neuroprotection against stroke and BBB disruption. E-A/P-CeO_2_ and A/P-CeO_2_, ceria nanoparticles loaded with or without edaravone and modified with Angiopep-2 (ANG) and polyethylene glycol (PEG) on their surface; P-CeO_2_, ceria NPs decorated with PEG. (**A**) Schematic representation of the Angiopep-2 (ANG-targeting) of lipoprotein receptor-related peptide (LRP) on the brain capillary endothelial cells (BCECs) that promote E-A/P-CeO_2_ crossing the BBB into brain tissue for the treatment of stroke. (**B**) Cerebral uptake of ceria nanoparticles was conducted on healthy rats whose BBB was complete. ICP-OES was used to measure the concentrations of ceria nanoparticles in brain tissue following intravenous injection. E-A/P-CeO_2_ nanoparticles were successfully carried across the BBB into the brain by an ANG-targeting and LRP-receptor-driven transcytosis mechanism in vivo. The results in Panel B demonstrate the accumulation of ceria NPs in the brain region within 24 h compared with P-CeO_2_ (* *p* < 0.05). (**C**) A model of stroke, middle cerebral artery occlusion (MCAO), was injected with different concentrations of ceria nanoparticles. Cerebral infarct volume was measured after 24 h by triphenyl tetrazolium chloride (TTC) staining. The normal stained tissue is red, and the unstained infarction is white. The result shows a high infarct volume of the control group of saline injection by 45.6 ± 4.8%. In contrast, treatment with E-A/P-CeO_2_ caused a decrease in infarction up to 15.0 ± 4.1% at an optimal concentration of 0.6 mg/kg in a dose-dependent manner. The neuroprotective effect of ceria nanoparticles indicates that E-A/P-CeO_2_ is more potent than A/P-CeO_2_ and P-CeO_2_, suggesting that edaravone attenuates ROS scavenging coupled with a targeted effect, thanks to the ceria core system which enhanced the antioxidant potency for improved treatment in vivo. Adapted with permission from [112]. Copyright {2018} American Chemical Society.

Ceria nanoparticles have been broadly studied as nanoscale antioxidant systems [112]. An example is given in Figure 9. Ceria nanoparticles can mimic the function of the antioxidant enzymes SOD and CAT. In redox processes, the cerium species transforms between two possible valence states of +4 (oxidized) and +3 (reduced) [113,114]. Cerium oxide NPs with a crystalline fluorite-type lattice structure display oxygen vacancies that result from the loss of oxygen and its electrons. Thus, in addition to the ionic state change from Ce^3+^ to Ce^4+^, the nanoparticle’s stoichiometry may also switch from CeO_2_ to CeO_2−x_ [115]. As a result, CeONPs can interact with various free radicals and detoxify their harmful action based on modifications in the redox state and oxygen vacancies.

In recent years, in vivo and ex vivo models have been used to assess the efficacy of nanoceria using an electrochemical biosensor based on cytochrome C [116]. In particular, it has been demonstrated that cerium oxide nanoparticles with a diameter of about 15 nm exhibit a SOD-like activity equivalent to 527 U of SOD for each 1 μg/mL nanoceria, being able to lower superoxide levels in a mice brain slice [116]. By using mouse hippocampus brain slices as an ex vivo model of ischemia, Estevez et al. conducted another interesting study that demonstrated how cerium oxide nanoparticles could lower cell death levels by 50% [117]. For the treatment of neurotrauma, Yan et al. fabricated a nanozyme based on Pt/CeO_2_ with effective catalytic activity. In vivo tests have shown that these species can accelerate healing and lessen neuroinflammation [118].

Additionally, CeONPs can regenerate their redox-active matrix, enabling repeated free-radical interactions, thanks to the lattice structure and simplicity of electronic conversions with other ionic species at the quantum level, allowing repetitive elimination of ROS [119].

Ceria nanoparticles have also shown a variety of therapeutic potentials for neuro- and cardio-protection. Due to their high surface-to-volume ratio, small ceria nanoparticles (<5 nm), particularly, have shown improved therapeutic effectiveness. Kim et al. demonstrated that 3 nm ceria nanoparticles might protect the brain against ischemic stroke in rats [120].

In another work, Kwon et al. successfully synthesized conjugate triphenylphosphonium ceria nanoparticles (TPP-ceria NPs) [119]. They demonstrated effectiveness by localizing to mitochondria and preventing neuronal degeneration in a 5XFAD transgenic mouse model of Alzheimer’s disease. In addition, TPP-ceria NPs also prevented reactive gliosis and morphological mitochondrial damage in mice [119].

### 4.2. Iron Oxide Nanoparticles in Regenerative Treatments

It has been suggested that iron oxide NPs may ameliorate neurodegeneration by mimicking the CAT activity and decomposing ROS [121,122]. Considering that significant microglial activation occurs prior to the creation of tangles in neurodegenerative tauopathies, reducing tau’s ability to trigger this activation has been demonstrated to slow the development of the pathology [121,123]. Glat et al. have shown that utilizing fibrin γ^377−395^ peptide conjugated to iron oxide (γ-Fe_2_O_3_) nanoparticles, with a diameter of 21 ± 3.5 nm, specifically, inhibits microglial cells in rTg4510 tau-mutant mice. The number of neurons with hyperphosphorylated tau and tangles was significantly reduced compared with untreated animals [124].

Other studies have addressed the impact of magnetic nanoparticles on Aβ fibrillation. Specific surface-coated superparamagnetic iron oxide nanoparticles (SPIONs) can interact with amyloid-beta (Aβ) and other amyloidogenic proteins. Moreover, in a magnetic field, SPIONs may be transported to the target tissue and may also be impacted by the applied field. It has been reported that the Aβ fibrillation process is suppressed at lower concentrations of nanoparticles and accelerates in the presence of a magnetic field and high concentrations of nanoparticles [125].

As a prospective treatment for neurodegenerative disorders, Katebi et al. demonstrated that combining nerve growth factor (NGF), quercetin, and superparamagnetic IONPs boosted neurite outgrowth and improved neurite branching in PC12 cells [126]. Consistent with these data, Chung et al. showed that the therapeutic effects of human mesenchymal stem cells (hMSCs) in a mouse model of PD, induced by a local injection of 6-OHDA, may be enhanced by dextran-coated iron oxide nanoparticles (Dex-IO NPs) [127]. In situ analyses have revealed that Dex-IO NPs may enhance the rescue impact of hMSCs on host DA neuron loss. Moreover, the data have shown that Dex-IO NPs can augment the ability of hMSCs to migrate toward lesioned DA neurons and drive hMSCs to differentiate into DA-like neurons at the disease site [127]. IONPs are also extensively explored in diagnostics and the treatment of neurodegenerative diseases, including AD, PD, and ALS. They are used as drug carriers and MRI contrast agents in AD, allowing the development of a multifaceted approach for targeting, diagnosing, and treating CNS disorders [121,128].

### 4.3. Manganese-Based Nano-Antioxidants

Aside from selenium, manganese (Mn) is another micronutrient that plays a crucial role in brain function because it can pass both the blood–brain barrier (BBB) and the blood–cerebrospinal fluid barrier (BCB). Furthermore, Mn^2+^ ions typically concentrate in the mitochondria of brain cells through divalent metal transporters. Manganese oxides have applications in the targeted imaging of neurodegenerative disorders [129]. Among the many 3D-transition-metal oxides, manganese oxides, such as MnO, Mn_2_O_3_, Mn_3_O_4_, Mn_5_O_8_, and MnO_2_, have attracted particular interest due to their wide range of structural and compositional variations [130,131]. Mn-oxide nanoparticles with good physicochemical properties hold a promise for sustainable nanotechnology developments. In particular, MnO_2_ has been explored for its ability to act as a nanoreactor in scavenging ROS [132,133]. In an acidic environment, MnO_2_ nanoparticles have a natural peroxidase-like activity that may break down H_2_O_2_ into water (H_2_O), oxygen (O_2_), and manganese ions (Mn^2+^) [134]. Some researchers have argued that MnO_2_ particles could occasionally increase oxidative stress rather than decrease it. Therefore, combining them with nanotechnology-based systems may aid in better controlling their release and toxicity, providing protection for cells and enhancing their distribution to the target tissues [135].

Kuthati et al. have reported that intrathecal treatment by manganese oxide nanoparticles (MONPs) dramatically reduced mechanical allodynia and the expression of COX-2, a crucial mediator of chronic and inflammatory pain in the spinal dorsal horns of PSNT rats [136]. In parallel, Singh and colleagues have demonstrated that the multienzyme mimic, Mn_3_O_4_ nanoparticles, provides exceptional protection to biomolecules against ROS-mediated protein oxidation, lipid peroxidation, and DNA damage, preventing cells from suffering oxidative damage [137]. In a separate study, Mn_3_O_4_ nanozymes demonstrated superiority to CeO_2_ nanozymes in ROS elimination. The effectiveness in vivo was evidenced by the prevention of ROS-induced ear inflammation in live mice [138]. Under hypoxic conditions, antioxidant manganese nanoparticles reduce free-radical load and improve oxygenation [139]. Based on these properties, MONPs provide a promising platform for developing metal nanoparticles as redox-active nanozymes to combat oxidative stress and inflammatory responses associated with SARS-CoV-2-related neurological pathologies.

### 4.4. Selenium and Nanoselenium

Selenium is a micronutrient that supports mammalian redox biology by maintaining normal intracellular ATP and Ca^2+^ homeostasis [97]. Food sources of selenium include grains, nuts, vegetables, seafood, meat, and dairy products [140,141]. Numerous epidemiological studies have shown a link between low levels of selenium and an increased risk of developing various pathologies, including cancer, neurodegenerative disorders, cardiovascular problems, and infectious diseases. Most of the beneficial effects of selenium result from its incorporation as selenocysteine into selenoproteins, a vital class of proteins. Selenocysteine is the 21st proteinogenic amino acid encoded by a UGA codon, which is usually the signal for the termination of protein synthesis [141]. Therefore, selenoproteins play a crucial role in the antioxidant defense mechanisms that maintain redox homeostasis, together with CAT, SOD, GSH, vitamin E, GPx, thioredoxin reductase, carotenoids, and ascorbic acid [142,143]. Recently, selenium levels have been found to favorably connect with COVID-19 survivors compared with non-survivors [144]. This hypothesis was supported by the reported lower selenium levels in COVID-19 patients (69.2 ± 8.7 ng/mL) compared with the controls (79.1 ± 10.9 ng/mL) [144]. Of note, Moghaddam et al. have established a clear correlation between the mortality in patients with COVID-19, low selenium levels, and selenoprotein P (SELENOP) [145].

Because selenium may accumulate in tissues and have lethal effects at high dosages on healthy tissues, nanoscale selenium (selenium nanoparticles, SeNPs) may be a highly efficient formulation with enhanced antioxidant activity and lower toxicity for targeted delivery in various pathologies, especially in viral infections. Studies have revealed that SeNPs efficiently protected C2C12 cells from H_2_O_2_ exposure by suppressing ROS and promoting myogenic differentiation. The latter is accompanied by an elevation of myogenic-related mRNA levels (MyoD, MyoG, and α-actinin), which promote multinucleated mature myoblasts and enhance the production of antiapoptotic proteins (e.g., BCL-2) [146]. Moreover, selenium nanoparticles stabilized with chitosan (Ch-SeNPs) have been reported to inhibit the Aβ_42_ aggregation produced by some amino acid enantiomers [147]. In the Hepatitis B infection, the delivery of a SeNPs/HBsAg vaccine in vivo impacted lymphocyte proliferation, elevated IFNγ levels, and triggered a Th1 response [148]. Thus, SeNPs may be a highly efficient therapeutic strategy against SARS-CoV-2 by enhancing humoral immune responses [148].

### 4.5. Other Inorganic Antioxidant Nanomaterials and Carbon-Based Nanomaterials

Platinum-based nanomaterials have been fabricated as another class of NPs with fascinating outcomes as nano-antioxidants that are able to mimic enzymatic CAT and SOD activities. Takamiya et al. have utilized Pt nanoparticles as a preventative approach to lessen the effects of ischemic stroke and to repair damages, while maintaining the structure and neurological capabilities of the neurovascular unit (NVU) in a mouse model of cerebral infarction [149]. Mu et al. have developed a trimetallic (triM) nanozyme with a multienzyme-mimetic activity that functioned as an effective scavenger of ROS and RNS in brain traumatic injuries [150].

Furthermore, yttrium oxide nanoparticles (Y_2_O_3_) have been reported as a neuroprotector in HT-22 mouse hippocampal neuronal cells in a rat model of lead-induced neuronal damage and an in vivo model of photo degeneration [151].

Several carbon-based nanomaterials, including fullerene, graphene nanosheets, carbon nanotubes, and carbon clusters, have been investigated as antioxidants and as possible treatments for some CNS disorders [152,153].

Two-dimensional carbon-based nanomaterials have also demonstrated antioxidant properties as indicated in the work of Qiu et al., who used electron paramagnetic resonance spectroscopy (EPR) to examine graphene’s capability to scavenge ROS [154]. The authors found that graphene oxide was able to do so for both OH and O_2_ radicals.

Altogether, these findings could lay the basis for the future exploration of both organic- and inorganic-based nanodelivery systems in post-COVID-19 neurological diseases characterized by high levels of oxidative stress.

## 5. Nanodelivery of Antioxidant Enzymes

To combat oxidative and nitrosative stress, cells employ a defense system including the superoxide dismutase (SOD), catalase (CAT), and glutathione peroxidase (GPX) antioxidant enzymes. Regarding the nanosystems for the delivery of antioxidant enzymes, the ability of the combination of NPs and tissue plasminogen activator (t-PA + nano-SOD/CAT) to stimulate the migration of stem/progenitor cells from the subventricular zone and circulation, and thus to promote neurogenesis, has been recently emphasized [155]. The inhibition of edema formation has suggested the protection of the BBB from reperfusion injury in a thromboembolic rat stroke model [155]. Another study has reported a significant reduction in mitochondrial ROS activities, increased mitochondrial membrane potential, reduced calcium levels, and also higher adenosine triphosphate (ATP) content after the intravenous administration of nano-SOD/CAT, 6 h after injury in a rat severe contusion model of spinal-cord injury (SCI), thus protecting cell apoptosis and further degeneration [156].

A summary of the outcomes of antioxidant-based nanoparticles and nanoconjugates with the potential to improve the neurological consequences of COVID-19 is presented in Table 1.

## 6. Conclusions

Current evidence on the prevalence of post-COVID-19 symptoms and the neurological cost of COVID-19 is a wake-up call for effective treatments. Indeed, substantial documentation buttresses the hypothesis that oxidative stress plays a crucial role in disease progression. Using antioxidants to scavenge free radicals appears to be an approach towards the right direction. Although antioxidants have limitations, nanotechnology-based drug-delivery systems can serve as a tool to combat such drawbacks. Nanotechnology allows the combination of different antioxidant agents in nanoscale reservoirs to improve their delivery, efficacy, and bioavailability. Furthermore, several phytochemicals may have synergistic antiviral effects when co-administered, and thus show superior efficacy in improving the clinical outcomes of neurological long COVID. Considering that oxidative stress is a common pathophysiological process in multiple diseases, an effective antioxidant nanomedicine-based approach could have broad therapeutic applications in many clinical settings.

## Figures and Tables

**Table 1:** Antioxidant-based nanoparticles and nanoconjugates with the potential to improve the neurological consequences of COVID-19.

	Nanosystem	Neurological Condition	Model/Administration Route	Outcome	References
**Organic NPs**	PLGA-PEG/Curcumin nanoparticle conjugate with B6 peptide	AD	In vitro: HT22 cells In vivo: APP/PS1 Al transgenic mice, intraperitoneal injection (IP)	○ Improved spatial learning and memory capability of APP/PS1 mice. ○ Reduced hippocampal Aβ formation and deposit. ○ Reduced tau hyperphosphorylation.	[66]
	Solid lipid curcumin (SLC)	Impaired cognition and mood	Healthy adults aged 60–85 y, oral administration	○ Improved performance on sustained attention, working memory tasks, and mood. ○ Significantly reduced total and LDL cholesterol.	[157]
	Curcumin-loaded nanocapsules (NLC Cs)	AD	In vivo: mice model of AD, gavage administration	○ Reduced Aβ-generated oxidative stress in the prefrontal cortex. ○ Increased SOD and CAT activities.	[158]
	Edaravone-loaded NPs	Cerebral hemorrhage	Edaravone injection (25 mg) to patients	○ Improved neurological function. ○ Reduced edema. ○ Reduced production and release of interleukin and tumor necrosis factor.	[159]
	Nanostructured lipid carriers (NLCs) containing resveratrol (NR)	Ischemic stroke	In vivo: rat model of middle cerebral artery occlusion (MCAO), IP injection	○ Reduced infarct volume. ○ Improved motor and cognitive function. ○ Ameliorated neuroinflammatory and oxidative-stress markers. ○ Enhanced activities of antioxidant enzymes and Na+, K+, ATPase. ○ Attenuated activities of caspases 3 and 9, IL-1β, IL−6, and TNF-ɑ.	[160]
	Dendrimer-based N-acetylcysteine (NAC)	Neuroinflammation, cerebral palsy (CP)	In vivo: CP rabbit model, IV injection	○ Suppressed neuroinflammation. ○ Improved motor function. ○ Improved myelination and attenuated neuronal injury. ○ Suppressed pro-inflammatory microglia.	[92]
	Bilirubin nanomedicine (BNM)	Cytokine storm syndrome	In vivo: IV administration	○ Halts cytokine storm by modulating the expression of the TGF-β, MAPKs, and NFκB signaling pathways.	[161]
**Inorganic NPs**	Lenalidomide and nanoceria (CeO_2_)	Autoimmune encephalitis	In vivo: C57BL/6 mice, IP injection	○ Eliminated experimental autoimmune encephalomyelitis (EAE) symptoms. ○ Reduced white-matter pathology and inflammatory responses.	[162]
	Endaravone-loaded ceria NPs (E-A/P-CeO_2_)	Stroke	In vitro: brain capillary endothelial cells (BCECs) In vivo: MCAO rat model, IV injection	○ Effective BBB crossing via receptor-mediated transcytosis. ○ Elimination of ROS.	[112]
	Nanoceria (CeO_2_)	Cytokine storm, mild brain injury	In vitro: mixed organotypic neuronal cultures In vivo: rat model of mTBI, injection	○ Suppressed inflammatory pathways (MAPKs, NF-κB, STAT3, iNOS, and COX) for the prevention of multiple-organ damage. ○ Enhanced cognitive recovery and motor performance. ○ Reduced neuronal death and calcium dysregulation.	[163,164]
**Antioxidant enzymes**	SOD1/CAT bioenzyme NPs	CNS delivery	In vivo: male Balb/c mice, intravenous	○ Increased stability of enzymes in both blood and brain.	[165]
	SOD1 cl-nanozymes	Ischemic brain injury	In vitro: immortalized bovine brain microvessel endothelial cells containing a Middle T-antigen gene (TBMECs) and CATH.a neuronal cell line In vivo: MCAO model, IV injection	○ Scavenged ROS in cultured brain microvessel endothelial cells and central neurons. ○ Decreased ischemia/reperfusion-induced tissue injury. ○ Improved sensorimotor functions.	[166]
	SOD-loaded PLGA NPs Nano-CAT	Neuroprotection	In vitro: human fetal neurons	○ Enhanced protection of neurons from oxidative stress. ○ Reduced protein oxidation, DNA damage, mitochondrial membrane transition pore opening and loss of cell membrane integrity. ○ Restored neuronal morphology, neurite network and microtubule-associated protein-2 levels.	[167,168]

## Data Availability

The authors confirm that the data supporting the findings of this study are available within the article.

## Abbreviations

4-HIA—4-Hydroxyisophthalic acid; 6-OHDA—6-hydroxydopamine; 8-OHG—8-hydroxyguanosine; ACE2—angiotensin-converting enzyme 2; AchE—acetylcholinesterase; AD—Alzheimer’s disease; ATP—adenosine triphosphate; AuNPs—gold nanoparticles; Aβ—amyloid beta; BACE1—beta-secretase 1; BBB—blood–brain barrier; BCB—blood–cerebrospinal fluid barrier; BCL-2—B-cell lymphoma 2; cAMP—cyclic adenosine monophosphate; CAT—catalase; CCL2—chemokine ligand 2; CNS—central nervous system; COX2—cyclooxygenase-2; DCNs—dopamine-containing neurons; ETC—electron transport chain; EGCG—epigallocatechin gallate; ER—endoplasmic reticulum; GABA—gamma-aminobutyric acid; GCG—gallocatechin gallate; GPx—glutathione peroxidase; GSH—glutathione; HBsAg—hepatitis B virus surface antigen; H_2_O_2_—hydrogen peroxide; I/R—ischemia/reperfusion; IL-10—interleukin 10; INF-γ—interferon gamma; INOS—nitric oxide synthase; IONPS—iron oxide nanoparticles; LDL—low-density lipoproteins; MAP2—microtubule-associated protein 2; MAPK—mitogen-activated protein kinase; MBP—myelin basic protein; MCI—mild cognitive impairment; MDA—malondialdehyde; ME/CFS—myalgic encephalitis/chronic fatigue syndrome; MSCs—mesenchymal stromal cells; MSNPs—mesoporous silica nanoparticles; MRI—magnetic resonance image; mTOR—mechanistic target of rapamycin; NAC—N-acetylcysteine; nAChR—nicotinic acetylcholine receptors; NADPH—nicotinamide adenine dinucleotide Phosphate; NF-κB—nuclear factor kappa-light-chain-enhancer of activated B cells; NGF—nerve growth factor; NPs—nanoparticles; NVU—neurovascular unit; O2-—superoxide; PASC—post-acute sequelae of SARS-CoV-2; PCL—poly (ɛ-caprolactone); PD—Parkinson’s disease; PEG—polyethylene glycol; PET—positron emission tomography; PI3K/Akt—phosphatidylinositol 3-kinase/protein kinase B; PLA—polylactic acid; PLGA—poly(lactic-co-glycolic acid); PMN—polymorphonuclear neutrophil cells; PNS—peripheral nervous system; POTS—postural orthostatic tachycardia syndrome; PrD—prion disease; PVR—periventricular region; RMT—receptor mediated transporters; RNS—reactive nitrogen species; ROS—reactive oxygen species; RSV—respiratory syncytial virus; SAPK/JNK—stress-activated protein kinase/Jun-amino-terminal kinase; SARS-CoV-2—severe acute respiratory syndrome coronavirus 2; SA—sialic acid; SBB—spinal–blood barrier; SCI—spinal cord injury; SiNPs—silicon nanoparticles, SOD—superoxide dismutase; SLNs—solid lipid nanoparticles; STAT3—signal transducer and activator of transcription 3; SYP—synaptophysin, TGFβ—transforming growth factor beta; TfR—transferrin receptor; TNF-α—tumor necrosis factor.
